# Breaking Barriers: Empowering Cervical Cancer Screening with HPV Self-Sampling for Sex Workers and Formerly Incarcerated Women in Toronto

**DOI:** 10.3390/curroncol31120590

**Published:** 2024-12-17

**Authors:** Mandana Vahabi, Jenna Hynes, Josephine Pui-Hing Wong, Natasha Kithulegoda, Masoomeh Moosapoor, Abdolreza Akbarian, Aisha Lofters

**Affiliations:** 1 Mandana Vahabi, Lawrence Bloomberg Faculty of Nursing, University of Toronto, 155 College Street, Suite 130, Toronto, ON M5T 1P8, Canada; 2 Li Ka Shing Knowledge Institute, Unity Health-St. Michael’s Hospital, Toronto, ON M5B 1W8, Canada; 3 ICES, Toronto, ON M4N 3M5, Canada; aisha.lofters@wchospital.ca; 4 Daphne Cockwell School of Nursing, Toronto Metropolitan University, Toronto, ON M5B 2K3, Canada; jph.wong@torontomu.ca (J.P.-H.W.); masoomeh.moosapoor@torontomu.ca (M.M.); aakbarian@torontomu.ca (A.A.); 5 Maggies’ Toronto, Toronto, ON M3C 0C3, Canada; 6 Dalla Lana School of Public Health, Toronto, ON M5T 3M7, Canada; 7 Peter Gilgan Centre for Women’s Cancers, Women’s College Hospital, Toronto, ON M5S 1B2, Canada

**Keywords:** cervical cancer, screening, HPV-self sampling, sex workers, formerly incarcerated women, access to health, discrimination

## Abstract

Background: Although cervical cancer (CC) is highly preventable through appropriate screening methods like the Papanicolaou (Pap) test, which enables early detection of malignant and precancerous lesions, access to such screening has not been equitable across social groups. Sex workers and people with records of incarceration are among the most under-screened populations in Ontario. Little is known about the acceptability and feasibility of HPV self-sampling (HPV-SS) as an alternative cervical cancer screening method for these groups. This online, community-based mixed-methods pilot study aimed to address this knowledge gap. Methods: Eighty-four under- and never-screened sex workers and ex-prisoners aged 25–69 years and residing in the Greater Toronto Area, were recruited by community peer associates. Participants completed an online survey and viewed short videos about CC and screening with Pap and HPV-SS. Those who opted for HPV-SS conducted the test at one of two collaborating organizations. Results: The median age of participants was 36.5 years. Most had limited knowledge about CC and screening. Approximately 13% identified as non-binary, and 5% as two-spirit or trans men, with the majority having completed secondary education. Of the participants, 88% chose HPV-SS, and one-third tested positive for high-risk HPV types. The ability to self-sample without judgment from healthcare providers was noted as a key advantage. However, there was a need for training on proper HPV-SS techniques. Conclusions: To improve cervical cancer screening among sex workers, increasing awareness through participatory community co-creation of sexual health education is essential. Additionally, offering HPV-SS as a screening option is crucial, given its demonstrated acceptability and feasibility within this population, many of whom lack a primary care provider and face discriminatory attitudes in healthcare settings.

## 1. Introduction

Human papillomavirus (HPV), the most common sexually transmitted infection (STI), is the primary cause of cervical cancer (CC), which has been the fastest-growing cancer in Canada (+3.7% per year) since 2015 [1]. Risk factors for HPV exposure include early sexual activity (before 16 years old), multiple sexual partners, co-infection with other STIs like HIV, smoking, and high parity (having three or more children). These factors, along with low socioeconomic status, significantly increase the risk of developing and dying from cervical cancer [2,3,4,5,6,7]. CC ranked among the top five cancers diagnosed in women aged 25–44 in Ontario [2]. In Canada, cervical cancer continues invading and taking women’s lives with recent estimates of 400 cervical cancer-related deaths and 1600 new cervical cancer diagnoses in 2024 alone [2]. Similarly, current estimates in Ontario, one of the most populated provinces in Canada, indicate that 660 women will be diagnosed and 150 will die from cervical cancer in 2024 [8].

Cervical cancer is highly preventable through appropriate screening, such as the Papanicolaou (Pap) test, which allows for the early detection of malignant and precancerous lesions. Despite this, participation in screening remains disappointedly low among structurally marginalized women and individuals with a cervix, including those who are racialized immigrants and refugees, Indigenous people, members of 2SLGBTQ+, sex workers, and ex-prisoners. These groups face numerous structural and individual barriers, including racism, gender expression discrimination, language barriers, lack of healthcare access, cultural stigma surrounding STIs, including HPV, transportation costs, limited knowledge of cervical cancer screening, and demanding life priorities [9,10,11,12,13,14,15].

Our study focused on sex workers and ex-prisoners, who are not only at elevated risk for HPV but also encounter multiple institutional and systemic barriers. These include stigma, cultural taboos surrounding sex work and incarceration, discriminatory and judgmental attitudes by healthcare providers, as well as logistical issues like service availability (e.g., no family physician, inconvenient clinic hours, long wait times). The persistence of these disparities suggest that innovative methods are needed to address these barriers and improve screening rates among equity-deserving groups like sex workers and ex-prisoners [15,16,17,18,19].

HPV self-sampling (HPV-SS) as a primary screening method has the potential to overcome these barriers [20,21,22,23,24,25,26,27]. HPV-SS is an easy, user-friendly tool that has shown high acceptability among diverse groups of women [27,28,29,30,31,32,33]. Women are taught to use swabs to self-collect vaginal samples that are analyzed for high-risk HPV strains responsible for causing CC. This alternative method of screening can be done at a woman’s chosen time and location with no third parties involved. Furthermore, it provides a sense of empowerment for those who are often excluded due to social stigma surrounding their social location and identity. There is solid evidence of the validity and acceptability of HPV-SS compared to clinician-collected cervical samples [34,35,36]. The HPV-SS kits have been tested successfully among non-responders in Europe and in Canada among under-housed women in British Columbia, and under or never screened (UNS) women in Manitoba and Ontario [30,31,37,38,39].

Guided by these findings, our community-based mixed-methods pilot study aimed to assess the accessibility and feasibility of HPV self-sampling for UNS sex workers and ex-prisoners in the Greater Toronto Area (GTA) in Ontario. This paper reports only the findings from the quantitative component of the study.

## 2. Methods and Materials

The quantitative component of our pilot community-based mixed methods study involved the recruitment of 84 sex workers and ex-prisoners through community centers serving this population in the Greater Toronto Area (GTA) (i.e., Maggie’s Toronto—A sex workers support organization and PASAN—a community-based prisoner health and harm reduction organization).

The study protocol was reviewed and approved by Toronto Metropolitan University (REB# 2023-165) and University of Toronto (REB# 45488).

Eligible participants were recruited by two peer associates/community champions identified by our collaborating community centers. They received a half-day Zoom training from our research team, covering the study protocol, eligibility criteria, ethical considerations, information about cervical cancer risk factors and screening, and HPV-SS.

The study involved two groups: participants who agreed to use the HPV-SS kit and those who did not. The inclusion criteria were as follows: aged 25–69; self-identified as a current or former sex worker and/or having a history of incarceration; self-reported time since the last Pap test was >4 years (including no history of a Pap test); no prior history of cervical cancer or hysterectomy; not pregnant or having given birth in the last 3 months; residing in the GTA; able to read, write, and communicate in English; able to provide informed consent; and willing to share contact information with the study team. Study participants were recruited from March 2024 to June 2024.

Study participants were approached by trained peer associates/community champions at the selected community centers (Maggie’s Toronto and PASAN). As trusted community members, they discussed the study with potential participants and assessed eligibility. Eligible individuals who agreed to be contacted were referred to our Research Associate (RA), a trained nurse. The RA followed up via phone or zoom to provide further information and address any questions. Those interested in participating and with computer access were emailed a link to review the online consent form. Participants without computer access were invited to use designated computers at the community centers.

After providing consent, participants were granted access to an online self-administered questionnaire. The survey collected information on sociodemographic and clinical characteristics, sexual health practices, knowledge of cervical cancer screening (Pap tests and HPV), and experiences of stigma related to sex work or incarceration in medical and social contexts. It also assessed their intention to use HPV-SS and perceived facilitators and barriers to accessing HPV-SS. The questionnaire took approximately 30 to 45 min to complete.

Upon receiving notification of completed questionnaires, the RA reviewed participants’ responses about their intention in undertaking HPV-SS. The RA then contacted those interested in HPV-SS to arrange a meeting at one of the two collaborating centers for them to conduct HPV-SS.

All participants were provided links to cervical cancer screening educational materials and short videos produced by Cancer Care Ontario, and Ontario Health that included:

- Cancer Care Ontario resources
- The Right Time for the Pap Test brochure
- Videos from Ontario Health: Hamilton FHT

Participants were then divided into two self-selected cohorts: Cohort A: Participants willing to use the HPV-SS and Cohort B: Participants who did not agree to use the kit but consented to participate in the study.

Participants in Cohort A were provided with a link to a short instructional video on how to collect their cervicovaginal specimen, produced by BC Cancer (HPV Test educational video). Members of this cohort were instructed to pick up the HPV-SS device (a dry Copan swab FLOQSswab^®^ 552C.80, Copan Italia S.P.A., Brescia, Italy) at one of the collaborating centers, where our Research Associate (RA) was present.

At the center, participants received an instruction sheet with a diagram illustrating the steps to obtain a cervicovaginal specimen. They were instructed to insert the swab intravaginally, rotate it three times, and collect the specimen. Participants could collect the sample in the nearest washroom or private room at the center, then place the swab in the provided tube and return it to the RA.

Once the sample was returned, the RA suspended the dry swab in a vial containing the medium (Roche Cell Collection Medium, Roche Molecular Systems, Inc., Branchburg, NJ, USA). The vials were then transported to a designated hospital microbiology lab in Toronto by the RA for analysis on the Roche cobas^®^ 6800 System using the cobas^®^ HPV test (Roche Molecular Systems, Inc., Branchburg, NJ, USA).

Test results were emailed directly to the RA and the first author, who then informed the participants of their results, as well as their primary care provider if contact information was provided and they were willing for us to share the results with their primary care providers.

- Negative Results: Participants with negative results received a letter from the research team, advising them to follow provincial guidelines and undergo routine screening in 5 years. If a primary care provider’s contact information was provided, they also received a notification.
- Positive Results: Participants with positive results received a letter advising them to follow up with their primary care provider for a Pap test. A member of the research team also called them directly. If a primary care provider’s contact information was provided, they were sent a letter.

Participants with positive results who did not have a primary care provider or preferred not to see their regular provider were assisted in arranging a visit at a community health center collaborating with our two community partners. They were instructed to bring their results letter. Additionally, participants were connected with the HealthCareConnect program to help them find a long-term primary care provider if they were interested.

Participants in Cohort B were also provided with a link to the instructional video on collecting their cervicovaginal specimen, in case they reconsidered their decision. All participants were compensated $70 for their time, internet usage costs, and travel expenses to the collaborating centers for HPV-SS.

## 3. Data Analysis

Participants completed online surveys via Qualtrics, which were subsequently exported to IBM SPSS Statistics version 28.0 for analysis. Descriptive statistics (e.g., frequencies, means, medians, modes, and standard deviations) were calculated to summarize sociodemographic and clinical characteristics, knowledge of cervical cancer and screening, perceived stigma related to sex work and incarceration, as well as intentions, perceived facilitators, and barriers to using HPV-SS.

For knowledge about cervical cancer and screening with Pap and HPV, an overall score was calculated by summing correct responses. Correct answers were assigned a value of 1, while incorrect or “don’t know” responses were assigned a value of 0. Each scale consisted of eight questions, with total scores ranging from 0 to 8. For self-reported perceived stigma during medical and social encounters, a five-point Likert scale was used across several domains. The overall score was calculated by summing the responses, where “strongly disagree” was assigned a score of 1 and “strongly agree” a score of 5. The scale included 10 questions, with participant scores ranging from 10 to 50.

## 4. Results

### 4.1. Section A: Participants Sociodemographic and Clinical Characteristics

#### 4.1.1. Sociodemographic Characteristics

**Figure 1:**
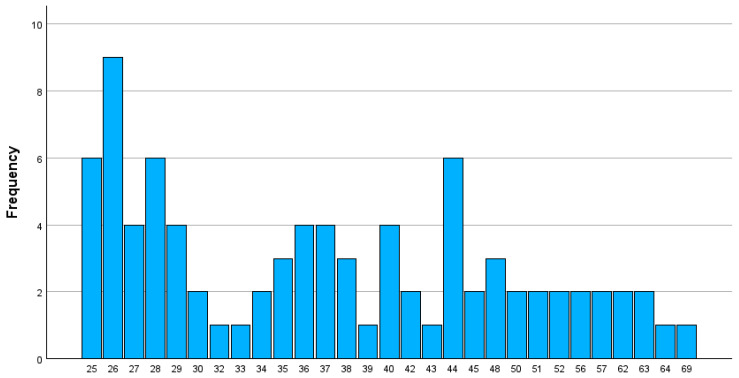
Age (Years).

The study surveyed 84 participants, with 40 recruited by Maggie and 44 by Pasan. Participants’ ages ranged from 25 to 69 years with a median age of 36.5 years and a standard deviation of 11.6 years. The age distribution was positively skewed (skewness = 0.76), indicating a younger cohort compared to the general population. Most participants were 45 years or younger (See Figure 1).

Approximately 80% of participants (n = 67) identified their gender as women, 13% as non-binary, and 5% as two-spirit or transgender masculine/genderqueer. Additionally, two respondents chose not to disclose their gender identity. Nearly 66% of the participants were single or never married, and 16% were married. Additionally, about 46% of participants reported having children, with the number of children ranging from one to six.

Regarding place of birth, 46.4% of the participants were not born in Canada. Of these, 32% had been living in Canada for less than 5 years, 5% for between 5 and 9 years, and the remainder for more than 10 years. Although 26% of participants who were born outside Canada preferred not to report their place of birth, the region of origin for the remaining included Africa, East Asia, South Asia, West Asia, South America, and the Caribbean. Additionally, among those born outside Canada, 26% were refugee applicants, approximately 15% were landed immigrants or had obtained Canadian citizenship, and 5% were either temporary migrant workers or held visitor visas.

The majority of the participants completed some form of secondary education, with approximately 60% (n = 50) having completed a college or university education. Conversely, a small fraction, around 7% (n = 6), had education levels below high school (grade 9 or less).

Most participants have been involved in sex work (i.e., 91%) and 33% reported having a history of incarceration.

Table 1 indicates that 37% of participants have been working as sex workers for 3 years or less, while the remainder have been involved for a longer period.

Study participants were asked to identify the types of sex work they engage in. The most reported type was independent escorting, followed by working as sugar babies and survival sex work (see Table 2).

About half of the participants reported being unemployed in the past 3 months (Table 3).

#### 4.1.2. Clinical Characteristics

One third of participants (i.e., 33%) reported their health as poor to fair while about half of participants reported their health as good and only 18% as very good to excellent.

Common cancers reported among family members were breast cancer (n = 20), cervical cancer (n = 13), and colon cancer (n = 7) followed by other cancers (e.g., lung, liver, prostate, throat, bladder, brain, kidney, lymphoma, skin) (n = 15). About 8% (n = 7) of individuals reported having been diagnosed with breast, colon, or other cancers.

Approximately 50% of participants reported having their first sexual experience at age 16 or younger. About 25% indicated having had six or more sexual partners in the past 3 months. More than half of the participants did not regularly use condoms during sexual encounters, and nearly half had previously been diagnosed with an STI, ranging from chlamydia to gonorrhea and HIV/AIDS. Around 24% (n = 20) of participants reported experiencing symptoms such as abnormal vaginal discharge, sores or ulcers on the vagina, or pelvic pain in the past 6 months. Surprisingly, only 40% (n = 8) of those affected sought medical attention. Of those who did, half were subsequently diagnosed with an STI (see Table 4).

### 4.2. Section B: Access to Healthcare Services

**Figure 2:**
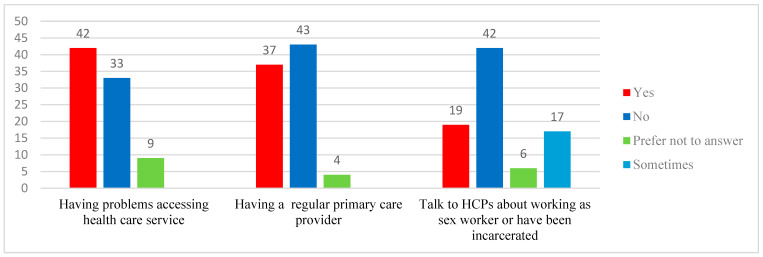
Difficulty accessing healthcare services and trust in disclosing personal information.

Half of the participants reported challenges in accessing healthcare services. Difficulties included long wait times to see family doctors, not having a regular family physician, lacking Ontario Health Insurance Plan (OHIP) coverage, and relying on walk-in clinics they believed were inadequately equipped to thoroughly address their health concerns or conduct follow-up tests. Participants also faced judgmental attitudes from healthcare providers and long delays in accessing mental health services. Many struggled to find healthcare providers who were sex-worker-friendly, 2SLGBTQ+ inclusive, and committed to anti-racist practices. Notably, about half of the participants did not have a consistent primary care provider and felt uncomfortable disclosing their sex work or criminal history to healthcare professionals (see Figure 2).

It is noteworthy that individuals who did not report difficulty accessing healthcare services were often affiliated with community health organizations such as Hassle-Free Clinic, Women’s Health in Women’s Hands, the Black Coalition for AIDS Prevention (Black CAP), the Toronto People With AIDS Foundation (PWA), and Fairview Sexuality Clinics. Some emphasized their preference for these confidential community services, driven by concerns about potential prejudice related to their occupation. Additionally, others received care through Family Health Teams or maintained long-standing relationships with regular general practitioners. Interestingly, one participant shared that during her migration process, her immigration physician seamlessly referred her to a specialist, minimizing potential challenges. Furthermore, some participants reported maintaining good health and thus infrequently needing healthcare services. However, having access to the Ontario Health Insurance Plan (OHIP) facilitated their use of walk-in clinics when necessary.

### 4.3. Section C: Perceived Risk of Encountering Cervical Cancer

A quarter of participants (25%) perceived the risk of an average woman encountering cervical cancer as slim to nonexistent. However, a notable 43% expressed concerns about their own susceptibility to cervical cancer in the future. Additionally, a staggering 80% believed that their lives would be significantly impacted if they were to face a diagnosis of cervical cancer.

### 4.4. Section D: Knowledge of Cervical Cancer and Screening

#### 4.4.1. Cervical Cancer

The participants’ overall performance on the cervical cancer knowledge assessment, comprising eight items, varied widely, with scores ranging from 0 to 8. The Cronbach’s alpha reliability coefficient for our set of eight items was calculated to be 0.635, indicating an acceptable level of internal consistency and reliability. On average, participants attained a mean score of 4.3, with a standard deviation of 2.1. The median and mode scores were both 5. Analysis of the data revealed that the distribution of knowledge levels among participants was as follows: 48% exhibited a low level of knowledge (total score of 0–4); 37% demonstrated a moderate level of knowledge (total score of 5–6); and 16% displayed a high level of knowledge (total score of 7–8).

#### 4.4.2. Pap Test

The participants’ overall performance regarding knowledge of the Pap test as a method for cervical cancer screening, assessed through an eight-item scale, exhibited significant variability, with total scores spanning from 0 to 8. The Cronbach’s alpha reliability coefficient was calculated to be 0.641, indicating an acceptable level of internal consistency and reliability. On average, participants achieved a mean score of 4.7, with a standard deviation of 1.6. The median and mode scores were 5 and 6, respectively. Analysis of the data revealed a distribution of knowledge level among participants as follows: 43% demonstrated a low level of knowledge (total score of 0–4); 50% displayed a moderate level of knowledge (total score of 5–6); and 7% exhibited a high level of knowledge (total score of 7–8).

The findings indicated a notable gap in participants’ awareness regarding the recommended screening guidelines: 43% were unaware of the recommended starting age for Pap test screening, while 73% were unaware of the recommended age for discontinuing screenings. Additionally, about one-third of participants (32%) were unaware of the recommended frequency of Pap tests (every 3 years). Furthermore, a quarter of the participants incorrectly believed that Pap tests were only recommended for older women, and 43% erroneously thought that Pap tests could lead to cervical cancer infection.

#### 4.4.3. HPV Screening

Although HPV screening is not yet included in Ontario’s cervical cancer screening guidelines and is not covered by OHIP, it is still available for those who are willing to pay out of pocket. The participants’ overall performance in understanding the HPV test as a method for cervical cancer screening, assessed via an eight-item scale, showed considerable diversity, with total scores ranging from 0 to 8. The Cronbach’s alpha reliability coefficient for our set of eight items was calculated to be 0.625, indicating an acceptable level of internal consistency and reliability. On average, participants achieved a mean and median score of 4.5, with a standard deviation of approximately 2. The mode score was 4.0. Analysis of the data revealed distribution of knowledge levels among participants as follows: 50% demonstrated a low level of knowledge, (total score of 0–4); 34.5% displayed a moderate level of knowledge (total score of 5–6); and 15.5% exhibited a high level of knowledge (total score of 7–8).

The findings underscored a notable gap in participants’ awareness regarding HPV testing for cervical cancer screening: 47% were unaware that individuals who have received the HPV vaccine still need to undergo HPV testing, while 79% were unaware that a positive HPV test result does not equate to an abnormal Pap test result. Additionally, approximately one-third of participants (34.5%) were unaware that the HPV test sample can be self-collected by them using the HPV self-sampling kit. Furthermore, more than two-thirds of the participants incorrectly believed that the HPV test can determine how long a woman has had HPV, and 43% erroneously thought that a positive HPV test does not mean an increased risk of developing cervical cancer.

### 4.5. Section E: Perceived Stigma During Medical Encounters or Other Social Interactions

Table 5 displays five-item Likert Scale responses ranging from strongly disagree to strongly agree from the study participants regarding self-reported stigma experienced during medical encounters or other social interactions. The Cronbach’s alpha reliability coefficient for our set of 10 items was calculated to be 0.85, indicating a high level of internal consistency and reliability.

The mean scores for items 4–5 and 8–10 exceed the weighted average, indicating that a considerable portion of participants reported internalized stigma and foresee enacting stigma from society, including their family and friends, due to their involvement in sex work or having a criminal record. Importantly, approximately a third of participants either agreed or strongly agreed that they had faced enacted stigma, such as being denied access to health services. Additionally, more than a third reported experiencing verbal harassment while receiving health or social care as well encountering judgmental attitudes from healthcare providers, characterized by negative remarks or gossip about them.

### 4.6. Section F: HPV-SS Uptake and Perceived Motivators and Barriers

#### 4.6.1. HPV-SS Uptake

Initially, among the 84 participants enrolled in the study, 77 showed interest in using the HPV-SS kit, while seven declined. Of the 77 participants who opted to use HPV-SS, three failed to attend their pre-scheduled testing appointments. Ultimately, among the 74 participants who successfully completed the test, 22 (29.7%) tested positive for HPV. Furthermore, 18% had high-risk HPV types 16/18.

#### 4.6.2. Perceived Motivators for Utilizing HPV Self-Sampling

The aspects of HPV-SS that resonated with participants and were captured in respondents’ responses to open-ended questions on the survey questionnaire included its enabling feature for women and individuals with a cervix to perform the sampling autonomously, without the presence and judgmental attitudes of health care providers. The accessibility of self-testing was highlighted as a boon, particularly for individuals with limited healthcare access or those who felt unprotected by their providers, providing them with a safe space and greater control. For those without regular health care providers, self-testing offered a convenient alternative, allowing them to take charge of their health and seek treatment as necessary.

Privacy and dignity were significant factors, with some feeling uncomfortable discussing sensitive topics with healthcare providers particularly when they had to disclose working as sex workers. Furthermore, traumatic or stressful experiences with traditional Pap tests led some to hope that the DIY (Do it Yourself) approach would be more comfortable and less judgmental, enabling them to listen to their bodies without pressure. Moreover, some participants highlighted that HPV-SS facilitates screening for individuals with a history of sexual or medical abuse, as well as those experiencing body dysphoria. The following quotes vividly illustrate the appeal of HPV-SS from the perspective of women and individuals with a cervix:

“I love the idea of being able to do the test in a private space. My previous Pap test was traumatic and extremely stressful, so I hope the DIY [Do it Yourself] test will be more comfortable without a judgmental presence and the ability to take my time and listen to my body”.

(sex worker, Age 26)

“As a sex worker, there are several aspects of HPV test kits that I may find appealing: HPV test kits offer the convenience of testing for the virus in the privacy of my own home, without the need to visit a healthcare provider or clinic… Using an HPV test kit allows me to take control of my sexual health and make informed decisions about prevention and treatment. It empowers me to proactively monitor my HPV status and seek appropriate healthcare interventions if needed. Overall, the convenience, control, confidentiality, accessibility, and peace of mind provided by HPV test kits are likely to be key reasons why women, including sex workers like me, may appreciate and benefit from using them as part of their sexual health”.

(sex worker, Age 51)

“As a trans man, I think it’s more accessible to do the kit on your own especially if you’re body dysphoric and/or have history of sexual trauma”.

(sex worker, Age 28)

“Able to be in control of the process and not have another touching my body. And being able to seek care for a diagnosis as opposed to having well-to-do doctors trying to impose their values or expectations upon you during their diagnosis/care process”.

(sex worker, Age 34)

“It feels less invasive than having a healthcare practitioner do it. I am not against having a worker do it in and of itself, but I do not want to risk being paired with one who will judge me”.

(sex worker, Age 25)

#### 4.6.3. Potential Barriers in Using HPV-SS

In addition to confidence in conducting the test correctly and cost considerations, participants identified a few other challenges that can contribute to underutilization of HPV-SS. These included a lack of awareness about the risks associated with HPV infection and the importance of screening, accessibility issues for nonbinary individuals like trans men due to the misconception that they do not require screening, privacy concerns about self-administering a test for a sensitive issue like HPV while residing in crowded households, and fear of further being judged, blamed, and stigmatized as carriers and transmitters of HPV. Additionally, follow-up care, counseling, and treatment after receiving a positive HPV result were considered particularly difficult for those without a regular primary care provider. Lastly, lack of emotional and psychological support while waiting for the test results was highlighted.

### 4.7. Section G: Reasons for Not Opting for HPV-SS

The main reason for the seven participants who declined to use HPV-SS were as follows: Some expressed preference for healthcare professionals (HCPs) to perform the test due to their expertise and skills, and some felt unprepared or uncomfortable to conduct it themselves. Others mentioned they would consider self-sampling only if they received thorough explanations and instructions.

## 5. Discussion

Our study aimed to assess the feasibility of HPV-SS in increasing cervical cancer screening uptake among under-screened and never-screened sex workers, as well as individuals with a history of incarceration. We found that 88% of participants opted to use HPV-SS, with one-third harbouring high-risk HPV types. Our findings align with previous literature about the feasibility of this screening method for women and individuals with a cervix who lack access to regular cervical cancer screening due to systemic barriers [27,28,29,30,31,33]. For participants in our study who had avoided Pap tests due to fear of invasive procedures or had not undergone one for years because of a lack of a regular primary care provider, discomfort, or discriminatory attitudes in healthcare settings, self-sampling offered a less anxiety-inducing and more accessible alternative for cancer screening. Many participants felt that self-testing empowered them to take control of their sexual health, particularly those with a history of sexual or medical trauma, as it offered a private and anonymous option.

Moreover, we found that among those who tested positive, about a quarter of them carried high-risk HPV types 16/18, which are responsible for 74% of invasive cervical cancer cases among Canadian women [40]. An estimated 6.2% of Canadian women are believed to harbor cervical HPV types 16/18 at any given time [40]. Our findings suggest that this rate is nearly three times higher in our study population. The elevated prevalence of high-risk HPV types in the study population may be linked to factors such as multiple sexual partners, exposure to sexual violence, unstable living conditions, and a criminalized working environment; stigma within healthcare settings can further limit access to life-saving reproductive health services [2,3,4,5,6,7,9,10,11,12,13,14,15]. This highlights the critical need for alternative screening methods, such as HPV-SS, particularly for structurally marginalized individuals with a cervix who face discrimination in accessing life-saving reproductive health services. The fact that nearly half of our participants lacked a regular primary care provider—delaying early detection of cervical cancer and access to medical treatments that could safeguard their reproductive health—further underscores the importance of HPV-SS in improving access to these essential services. Additionally, the discriminatory attitudes of healthcare providers pose a significant barrier for marginalized groups attempting to access available healthcare resources. It is therefore vital to educate healthcare providers and foster safe, inclusive environments that support sex workers and gender-diverse individuals. These efforts can reduce stigma and ensure that all individuals feel comfortable accessing essential sexual and reproductive health care.

Despite many participants having a high level of education, nearly half demonstrated limited knowledge about cervical cancer and screening. For example, approximately one-third of participants were unaware of the recommended frequency for Pap tests, which is every 3 years. Additionally, nearly half (43%) believed that Pap tests could cause cervical infection. These findings underscore the urgent need for co-creating visual, accessible, and art-based reproductive health education tailored to the specific learning needs of sex workers and delivered in formats (e.g., infographics, graphic novels, plain language fact sheets with artwork) and spaces that are easily accessible to them. This approach not only overcomes literacy barriers, which is crucial for sex workers with varying levels of formal education, but also provides an inclusive and community-centred way to learn [41]. Such a format can be particularly important for those who may feel uncomfortable or unsafe in traditional healthcare settings.

It is noteworthy that approximately one-third of participants identified their sex work as “survival sex work”, which refers to engaging in sex work to meet basic human needs such as shelter, food, and clothing. This highlights the urgent need for adequate community resources and support services that address these fundamental needs, including access to housing, food security, and healthcare. Furthermore, additional research and advocacy are necessary to develop targeted interventions that can effectively address the social, economic, and health disparities experience by this systematically marginalized population.

We found that over 60% of participants did not use protection during sexual intercourse, which further increases their risk of exposure to HPV and other STIs, including HIV. Recognizing that sex workers frequently have limited power or ability to require clients to use condoms, it is crucial to provide free access to female condoms, dental dams, and lubricants as protective barriers against STIs. In Ontario, these items can be accessed at no cost or at a reduced cost through harm-reduction programs and services offered by community organizations, public health units, and sexual health clinics. Considering the Ontario government’s move towards closing certain harm-reduction services [42], the potential ramifications for underserved populations could be devastating, further widening the health inequity gap. However, our study did not investigate the reasons behind participants’ inconsistent use of protection during sexual intercourse or their awareness of existing harm-reduction services, highlighting the need for further research in this area.

Our study also found that limited or inadequate access to mental health resources was a significant concern, particularly among equity-deserving groups with a history of sexual abuse and violence. Additionally, some participants highlighted the anxiety and stress associated with undergoing testing and receiving a diagnosis. It is imperative to ensure access to psychological and emotional support services both before and after cervical cancer screening for these groups.

While this study contributes to the existing literature on the subject, several limitations should be noted when interpreting the results. First, the small sample size and the absence of probability sampling may hinder the generalizability of the findings to the broader population of sex workers and individuals with a history of incarceration. Although not ideal, the use of nonprobability sampling was necessary due to the lack of a sampling frame for our target population. Second, the online design of our study may have restricted access for many potential and eligible participants who lacked access to computers or the internet. Although we provided the option for participants to use a designated computer at our collaborating agencies, this may still have discouraged some individuals from participating. Lastly, it is important to note that the study excluded immigrants and refugees who could not read, write, or converse in English. This limitation highlights the need for future research to include a more diverse population to better understand the experiences and barriers faced by all individuals in accessing cervical cancer screening.

## 6. Conclusions

Our study contributes to the growing literature on cervical cancer (CC) screening barriers among sex workers and individuals with a history of incarceration, highlighting the feasibility of HPV HPV-SS as an effective method to increase cervical cancer screening uptake. With 88% of participants opting for HPV-SS and one-third testing positive for high-risk HPV types, our findings underscore the importance of addressing the systemic barriers that prevent marginalized populations from accessing essential health services. HPV-SS not only serves as a viable screening approach to dismantle long-standing structural barriers but also empowers individuals to take control of their sexual health, providing a means to access screening without exposure to discriminatory attitudes in healthcare settings.

## Figures and Tables

**Table 1:** Number of Years Working as Sex Worker.

# of Years	Frequency	Percent
Less than 1 year	7	9.2
1–3 years	21	27.6
3–4 years	12	15.8
5–9 years	15	19.7
10+ years	16	21.1
Prefer not to answer	5	6.6
Total	76	100.0

**Table 2:** Types of Sex Work.

Types	Frequency	Percent
An agency escort	8	9.5
A fetish worker (i.e., Pro-Domme/sub)	18	21.4
An independent escort	44	52.4
A massage parlour attendant	9	10.7
An outdoor worker	18	21.4
Working for someone else (i.e., pimp, a manager)	9	10.7
A stripper	12	14.3
A porn performer	6	7.1
A sugar baby	31	36.9
A webcam host	19	22.6
Survival sex work: (sex work in exchange for basic needs/necessities (e.g., for food, shelter/housing, transportation, substances and/or medication or things you needed)	25	29.8
Other-Please specify only fans team/phone sex	2	2.4
Prefer not to answer	4	6.6

**Table 3:** Type of Employment in the Past 3 Months.

Type of Employment	Frequency	Percent
House cleaner/nanny	3	3.6
Work in a bar, restaurant, or shack	3	3.6
Work in a public business	7	8.3
Work for a private business	1	1.2
Dancer or performance arts	7	8.3
Other (online sex work, sex work and bartending, PSW, Cam girl, sugar baby, harm reduction worker, student, community services)	21	25.0
Unemployed	40	47.6
Prefer not to answer	2	2.4
Total	84	100.0

**Table 4:** Sexual Health Characteristics N (%).

Having Engaged in Sexual Intercourse N (%)	
	84 (100)
**Age at first sexual intercourse**	
16 years old or less	41 (48.8)
Over 16 years old	39 (46.4)
Prefer not to answer	4 (4.8)
Total	84 (100)
**Number of partners in the past 3 months**	
**1**	**26** (**31.0**)
2–5	29 (34.5)
6–9	10 (11.9)
10 or more	11 (13.1)
Prefer not to answer	8 (9.5)
Total	84 (100.0)
**Condom use during sex**	
Always	10 11.9
Usually	22 (26.2)
Sometimes	27 (32.1)
Rarely	11 (13.1)
Never	10 (11.9)
Prefer not to answer	4 (4.8)
Total	84 (100.0)
**Being diagnosed with sexually transmitted infections** (**STIs**)	
Yes	43 (51.2)
No	35 (41.7)
Don’t Know	4 (4.8)
Preferred not to answer	2 (2.4)
Total	84 (100.0)
**STIs**	
Chlamydia	20 (23.8)
Hepatitis	3 (3.6)
Herpes	7 (8.3)
Gonorrhea	9 (10.7)
Syphilis,	8 (9.5)
HIV/AIDS	8 (9.5)
Pelvic inflammatory diseases	3 (3.6)
Other STIs (genital wart, HPV)	11 (13.1)
**Abnormal discharge from vagina/sore or ulcer near or on vagina/pelvic pain in the past 6 months**	
Yes	20 (23.8%)
Number of participants who saw a health care provider for these problems	8 (40%)
Number of people who were told to have a STI	4 (50%)

**Table 5:** Perceived Stigma During Medical Encounters or Other Social Interactions.

Items	SD (%)	D (%)	Neutral (%)	A (%)	SA (%)	Mean	SD
1. I do not seek healthcare because someone might learn that I am a sex worker or have been incarcerated.	16 (19.0)	21 (25.0)	21 (25.0)	20 (23.8)	6 (7.1)	2.75	1.22
2. I have been denied health services or had someone keep me from receiving health services because I am a sex worker or have been incarcerated.	13 (15.5)	34 (40.5)	15 (17.6)	17 (20.2)	5 (6)	2.61	1.15
3. I have been verbally harassed when receiving health or social care because I am a sex worker or have been incarcerated.	15 (17.9)	22 (26.2)	16 (19.0)	20 (23.8)	11 (13.1)	2.88	1.32
4. I feel excluded from my friends and family because I am a sex worker or have been incarcerated.	8 (9.5)	17 (20.2)	21 (25)	24 (28.6)	14 (16.7)	3.23	1.23
5. Negative remarks or gossip was made by a family member about me because I am a sex worker or have been incarcerated.	7 (8.3)	15 (17.9)	21 (25)	22 (26.2)	19 (22.6)	3.37	1.25
6. Negative remarks or gossip was made by a healthcare worker about me because I am a sex worker or have been incarcerated.	7 (8.3)	25 (29.8)	22 (26.2)	17 (20.2)	13 (15.5)	3.05	1.21
7. Working as a sex worker or having a history of incarceration makes me feel that I do not have value.	14 (16.7)	18 (21.4)	18 (21.4)	16 (19.0)	18 (21.4)	3.07	1.40
8. People’s attitudes about sex workers or people who have been incarcerated make me feel worse about myself.	10 (11.9)	15 (17.9)	16 (19.0)	14 (16.7)	27 (32.1)	3.46	1.46
9. I work hard to keep my work as a sex worker or having been in prison a secret.	12 (14.3)	12 (14.3)	17 (20.2)	17 (20.2)	26 (31.0)	3.39	1.42
10. It’s easier to avoid friendships than worry about telling others you are a sex worker.	10 (11.9)	15 (17.9)	19 (22.6)	20 (23.8)	20 (23.8)	3.30	1.33

Note: SD = Strongly Disagree, D = Disagree, Neutral = (Neither disagree nor agree), A = Agree, SA = Strongly Agree. Weighted Average = 3.11.

## Data Availability

Data supporting reported results can be found. All supporting data have been reported in the Section 4 of the paper.

## Abbreviations

CC	Cervical cancer
Greater Toronto Area	GTA
HPV	Human papillomavirus
HPV-SS	Human papillomavirus Self-Sampling
Pap test	Papanicolaou test
RA	Research Associate
STIs	Sexually transmissible/transmitted infections
UNS	Under or never screened
